# Beyond glycemic control: SGLT2 inhibitors as time-sensitive organ-protective agents in acute injury—mechanistic insights and translational implications

**DOI:** 10.3389/fphar.2026.1860779

**Published:** 2026-06-25

**Authors:** Ji Wu, Yaozheng Cai, Jiangtao Chen, Qian Zang, Ping Zhu, Yueping Ding

**Affiliations:** 1 Second Clinical Medical College, Zhejiang Chinese Medical University, Hangzhou, China; 2 Department of Intensive Care Unit, The Second Affiliated Hospital of Zhejiang Chinese Medical University, Hangzhou, China; 3 Department of Hospital Infection Management, The Second Affiliated Hospital of Zhejiang Chinese Medical University, Hangzhou, China

**Keywords:** SGLT2 inhibitors, acute organ injury, cardiorenal protection, acute heart failure, acute kidney injury, myocardial infarction, mitochondrial homeostasis, endothelial dysfunction

## Abstract

**Background:**

Sodium-glucose cotransporter 2 inhibitors (SGLT2is) were initially developed as glucose-lowering therapies for type 2 diabetes mellitus. However, large cardiovascular and renal outcome trials have demonstrated rapid reductions in heart failure events and slowing of kidney disease progression, including in patients without diabetes, suggesting benefits beyond glycemic control.

**Objective:**

To summarize the mechanistic, preclinical, and clinical evidence supporting SGLT2is as acute organ-protective agents and to evaluate their translational potential in time-sensitive clinical settings.

**Methods:**

A structured narrative review was conducted using PubMed, Web of Science, and Embase, focusing on mechanistic studies, experimental acute injury models, randomized controlled trials, *post hoc* analyses, meta-analyses, and real-world studies published predominantly within the last 5–7 years.

**Results:**

SGLT2is may exert acute organ-protective effects through integrated mechanisms involving improved cellular stress adaptation, modulation of ionic and metabolic homeostasis, and hemodynamic as well as immuno-endothelial regulation. Current clinical evidence is most consistent in acute heart failure and early post-myocardial infarction (post-MI) remodeling, whereas evidence in acute kidney injury arrhythmias, stroke, acute lung injury, and liver injury remains limited or exploratory.

**Conclusion:**

SGLT2is should no longer be viewed solely as glucose-lowering agents. Accumulating evidence supports their broader role as potential time-sensitive organ-protective therapies in acute illness, particularly within the cardiorenal spectrum. However, substantial heterogeneity in evidence quality persists, and dedicated acute-care trials are needed to clarify therapeutic timing, patient selection, and safety considerations.

## 1 Introduction

Sodium-glucose cotransporter 2 inhibitors (SGLT2is) were originally introduced as insulin-independent glucose-lowering agents for type 2 diabetes mellitus. By inhibiting glucose and sodium reabsorption in the proximal renal tubule (Li et al., 2024), these agents induce glucosuria and natriuresis while carrying a relatively low intrinsic risk of hypoglycemia (Aftab et al., 2020; Hsu et al., 2023). Over the past decade, however, the clinical profile of this drug class has evolved substantially. Large cardiovascular and renal outcome studies have shown that SGLT2is reduce heart failure (HF)hospitalization and delay progression of chronic kidney disease, including in patients without diabetes, thereby challenging the traditional view that they function solely as antihyperglycemic agents (Pac et al., 2023; Giorgino et al., 2020; Vaduganathan et al., 2022).

A particularly important observation is that the clinical benefits of SGLT2is often emerge early after treatment initiation. In several major trials, separation of event curves occurred within weeks rather than years, a timeline that is difficult to attribute to improved glycemic control alone (Vaduganathan et al., 2022; Voors et al., 2022; Wang and Isom, 2020). This has prompted increasing interest in whether SGLT2is may act as time-sensitive modulators of acute organ stress responses, rather than merely as chronic disease-modifying agents. Such a perspective is highly relevant because acute organ injury—including acute heart failure (AHF), acute kidney injury (AKI), myocardial infarction (MI), arrhythmias, stroke, acute respiratory distress syndrome (ARDS), and acute liver injury (ALI)—remains a major source of mortality and healthcare burden worldwide.

Several lines of evidence support this broader interpretation. Experimental studies suggest that SGLT2is modulate mitochondrial quality control, cellular energy sensing, ionic homeostasis, oxidative stress, inflammation, endothelial integrity, and microvascular function (Huang et al., 2023; Matuszewski et al., 2025; Lee and Riella, 2024; Packer et al., 2024; Zannad et al., 2022; Billing et al., 2024; Durante et al., 2021). These biological effects are directly relevant to the pathogenesis of acute tissue injury. At the same time, translational and clinical data increasingly suggest that SGLT2is may influence outcomes in selected acute settings, especially AHF and early post-MI remodeling (post-MI) (Voors et al., 2022; Song et al., 2025; Von et al., 2022).

Although previous reviews have summarized the chronic cardiorenal benefits of SGLT2is, a focused synthesis of their role in acute organ protection remains lacking. Existing literature often addresses individual pathways or isolated disease settings without integrating them into a coherent translational framework. Moreover, the strength of evidence differs markedly across organs, and this heterogeneity deserves explicit evaluation.

In this review, we propose that SGLT2is may be understood as multi-layer organ-protective agents in acute injury settings through coordinated mitochondrial, metabolic, hemodynamic, and immunovascular mechanisms. We summarize the biological basis of these effects, critically appraise organ-specific evidence, discuss safety considerations, and outline research priorities needed to advance the field.

## 2 Why acute benefits matter: clinical signals of early separation

A central reason to revisit SGLT2is in the context of acute organ injury is the reproducible observation of early clinical benefit. Unlike therapies whose advantages depend on prolonged modification of atherosclerotic burden or long-term remodeling, SGLT2is often show rapid reductions in worsening HF events and favorable cardiorenal signals shortly after initiation (Vaduganathan et al., 2022; Voors et al., 2022; Packer et al., 2020). This temporal pattern suggests that their benefits arise, at least partly, from mechanisms capable of acting on short timescales, such as decongestion, neurohormonal modulation, improvement in endothelial function, attenuation of tubular stress, and enhancement of metabolic resilience.

The case is further strengthened by the fact that benefit is not restricted to patients with diabetes. In HF and chronic kidney disease populations, outcome improvements have been seen regardless of glycemic status (Vaduganathan et al., 2022; Wang and Isom, 2020; Anker et al., 2021). If glucose reduction is not necessary for therapeutic benefit, then non-glycemic mechanisms must occupy a central role.

From a translational standpoint, this matters because acute injury evolves through rapid, self-amplifying loops: venous congestion worsens renal dysfunction; mitochondrial stress increases inflammation; endothelial disruption promotes edema and microvascular malperfusion; and maladaptive remodeling begins soon after ischemic injury. SGLT2is may interfere with several of these loops simultaneously. However, enthusiasm must be tempered by evidence hierarchy. AHF currently provides the strongest support; post-MI remodeling is increasingly persuasive; AKI prevention remains emerging; and stroke, ARDS, and ALI are still largely exploratory. A rigorous review should therefore distinguish plausible biology from established clinical utility.

## 3 Mechanistic framework of acute organ protection by SGLT2 inhibitors

### 3.1 Cellular stress resistance and mitochondrial quality control

Acute organ injury commonly converges on a shared cellular phenotype characterized by mitochondrial dysfunction, ATP depletion, excessive reactive oxygen species (ROS) generation, impaired organelle turnover, and activation of cell death programs. SGLT2is appear to modulate several of these core injury pathways.

**FIGURE 1:**
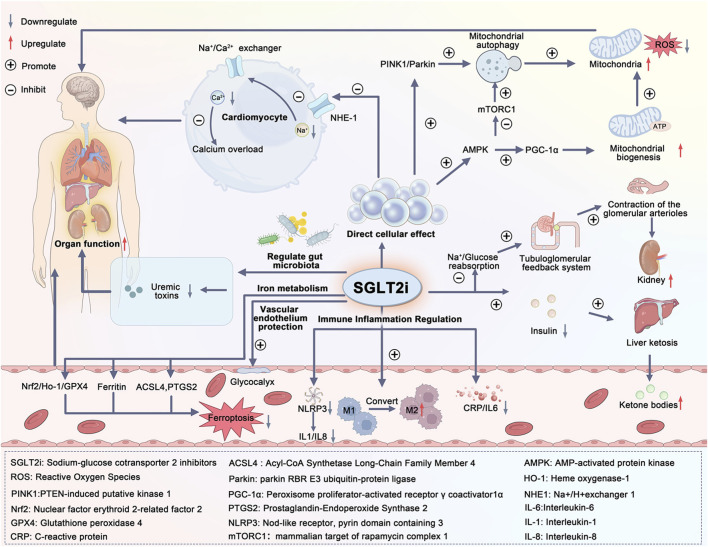
Mechanistic insights into acute organ protection mediated by SGLT2 inhibitors.

One of the most widely described mechanisms is activation of AMP-activated protein kinase (AMPK) with downstream suppression of mTORC1, thereby promoting autophagy (Hsu et al., 2023; Huang et al., 2023; Packer et al., 2024). This is relevant because autophagic flux enables cells to remove damaged proteins and dysfunctional organelles under conditions of ischemia, inflammatory stress, and metabolic overload. In the heart and kidney, where energy demand is high, such effects may improve tolerance to acute injury. Mitophagy, the selective autophagic elimination of dysfunctional mitochondria, appears particularly important and may involve pathways such as PINK1/Parkin signaling (Feng et al., 2025). By preserving mitochondrial quality, SGLT2is may limit oxidative stress amplification and reduce propagation of injury (Table 1, Figure 1).

**TABLE 1:** Mechanistic pathways underlying acute organ protection by SGLT2 inhibitors.

Mechanistic domain	Representative pathways	Functional consequence	Most relevant organs	Evidence level
Cellular stress resistance	AMPK activation, mTORC1 inhibition, autophagy, mitophagy	Improved organelle turnover and stress adaptation	Heart, kidney, liver	Moderate (preclinical)
Mitochondrial quality control	Reduced ROS generation, improved mitochondrial quality control	Preserved ATP production, lower oxidative injury	Heart, kidney, brain	Moderate (preclinical)
Ferroptosis modulation	Nrf2/HO-1/GPX4, ACSL4/PTGS2 modulation	Reduced iron-dependent lipid peroxidation	Kidney, heart, liver	Emerging (preclinical)
Ionic homeostasis regulation	NHE1 inhibition, reduced intracellular Na^+^, improved Ca^2+^ handling, CaMKII modulation	Less arrhythmia and reperfusion injury	Heart, possibly brain	Moderate (preclinical)
Metabolic reprogramming	Mild ketogenesis, altered substrate utilization	Improved energetic efficiency during stress	Heart, kidney, brain	Moderate (preclinical)
Renal hemodynamic effects	Restored tubuloglomerular feedback, reduced intraglomerular pressure	Lower filtration stress, improved renal resilience	Kidney	Strong (biological rationale)
Decongestive effects	Natriuresis with preferential interstitial fluid removal	Reduced preload, edema, and venous congestion	Heart, lung, kidney	Strong (clinical evidence)
Immunomodulati-on	NLRP3 inhibition, macrophage polarization	Lower inflammatory amplification, enhanced repair	Heart, kidney, brain, lung	Moderate (preclinical)
Endothelial barrier protection	Glycocalyx preservation, improved endothelial function	Better microvascular integrity, reduced vascular leak	Heart, kidney, lung, brain	Emerging (preclinical)

Strength of evidence was graded according to the consistency of findings across experimental and clinical studies, availability of randomized controlled trial data, reproducibility across independent cohorts, and degree of association with clinically relevant outcomes. “Strong” indicates consistent support from multiple randomized or large-scale clinical studies; “Moderate” indicates supportive mechanistic and clinical evidence with limited randomized validation; “Emerging” indicates predominantly preclinical, observational, or exploratory clinical evidence; and “Weak” indicates speculative or insufficiently validated evidence.

These effects may also be complemented by stimulation of mitochondrial biogenesis through AMPK/PGC-1α-related pathways, although the extent of this response likely varies according to tissue context and specific compound (Huang et al., 2023). In acute myocardial ischemia or ischemia-reperfusion injury, maintaining a healthier mitochondrial pool may attenuate ATP depletion, calcium overload, and subsequent contractile dysfunction (Table 1, Figure 1).

Another mechanistic dimension is the attenuation of oxidative stress. Improved mitochondrial efficiency, reduced cytosolic sodium overload, and blunting of inflammatory signaling collectively decrease ROS production (Huang et al., 2023; Matuszewski et al., 2025; Lee and Riella, 2024). Because ROS contribute to lipid peroxidation, DNA damage, endothelial dysfunction, and inflammasome activation, this effect may help explain the broad tissue-protective profile of SGLT2is (Table 1, Figure 1).

Increasing attention has also been directed to ferroptosis, an iron-dependent form of regulated cell death driven by lipid peroxidation. Experimental studies suggest that SGLT2is may influence pathways involving Nrf2/HO-1/GPX4, ferritin regulation, and markers such as ACSL4 and PTGS2, thereby reducing ferroptotic susceptibility (Zhang et al., 2025; Wu et al., 2021; Wang et al., 2022). Although direct evidence remains largely preclinical, this mechanism is attractive in AKI, myocardial ischemia, and hepatic injury, where iron-catalyzed oxidative injury has become a major topic of investigation (Table 1, Figure 1).

Taken together, these findings suggest that SGLT2is enhance intrinsic cellular resilience by improving energy sensing, promoting organelle quality control, decreasing oxidative stress, and modulating regulated cell death pathways. These effects are biologically coherent with the concept of acute organ protection independent of blood glucose lowering.

### 3.2 Ionic homeostasis and electrophysiological stabilization

Disrupted sodium and calcium handling is a hallmark of acute cardiac injury and contributes to contractile dysfunction, mitochondrial stress, arrhythmogenesis, and cell death. One proposed non-glycemic mechanism of SGLT2is is inhibition of the Na+/H+ exchanger 1 (NHE1) in cardiomyocytes, leading to lower intracellular sodium concentration (Aftab et al., 2020; Kolesnik et al., 2022). Reduced sodium accumulation may secondarily improve calcium handling through the sodium-calcium exchanger, thereby attenuating calcium overload and downstream injury (Table 1, Figure 1).

This mechanism is particularly relevant in myocardial ischemia-reperfusion injury and AHF. During ischemia, intracellular acidosis stimulates sodium influx, which then promotes calcium overload, mitochondrial swelling, and electrical instability. By improving ionic balance, SGLT2is may reduce susceptibility to ventricular arrhythmia and reperfusion injury. In addition, experimental data indicate that empagliflozin can inhibit late sodium current through modulation of CaMKII, offering another pathway by which electrical stability may be enhanced (Mustroph et al., 2022).

These observations are consistent with animal data showing attenuation of QT prolongation and reduced vulnerability to ischemia/reperfusion-induced arrhythmias (Özgür et al., 2021). Although the strongest evidence concerns the heart, analogous principles may also be relevant to neural tissue, where ionic imbalance and excitotoxicity contribute to acute ischemic brain injury.

Overall, ionic and electrophysiological stabilization represents an important mechanistic layer linking SGLT2is to acute myocardial protection, arrhythmia reduction, and possibly broader ischemic resilience.

### 3.3 Systemic metabolic reprogramming

SGLT2is induce a characteristic metabolic shift involving mild increases in ketone body availability, reduced circulating insulin, altered glucagon-insulin balance, and changes in substrate preference (Matuszewski et al., 2025; Santos-Gallego et al., 2023; Fatima et al., 2023). This metabolic profile is often interpreted as a form of systemic reprogramming that may improve energetic efficiency during organ stress.

Among these changes, β-hydroxybutyrate has received particular attention. Ketone bodies may serve as an efficient fuel source for the failing heart and other energy-deprived tissues, potentially generating ATP more efficiently per molecule of oxygen consumed than fatty acids (Santos-Gallego et al., 2023; Yurista et al., 2021). In addition to acting as fuels, ketones also serve as signaling molecules with anti-inflammatory and antioxidative properties, including inhibition of the NLRP3 inflammasome and modulation of histone deacetylase activity (Lee and Riella, 2024; Yurista et al., 2021). This dual bioenergetic and signaling role may be particularly relevant in acute states characterized by metabolic mismatch.

The kidney may benefit from metabolic reprogramming in a different way. By reducing proximal tubular sodium-glucose cotransport, SGLT2is decrease tubular transport workload and oxygen consumption, thereby improving the balance between oxygen demand and supply (Giorgino et al., 2020; Bailey et al., 2022). This effect may be especially valuable in diabetes, cardiorenal syndrome, and ischemic AKI, where tubular hypoxia is a key driver of injury.

A more speculative but intriguing area concerns the gut-organ axis. Emerging data indicate that SGLT2is may modulate gut-derived metabolic signals and reduce circulating levels of protein-bound uremic toxins such as indoxyl sulfate and p-cresyl sulfate, possibly through microbiota-associated effects (Billing et al., 2024; Hsu et al., 2025). Although causality and clinical relevance remain incompletely defined, such changes may contribute to systemic anti-inflammatory and vascular benefits (Figure 1).

Importantly, the metabolic effects of SGLT2is also carry risk. The same tendency toward ketosis that may support stressed organs can, under conditions of fasting, infection, perioperative stress, or insufficient insulin, predispose to euglycemic diabetic ketoacidosis (Wang and Isom, 2020). Thus, metabolic reprogramming is both a source of therapeutic promise and a major determinant of safety boundaries.

### 3.4 Hemodynamic unloading and cardiorenal coupling

The hemodynamic actions of SGLT2is are among the most direct explanations for their early clinical benefits. By inhibiting proximal tubular sodium and glucose reabsorption, these agents increase sodium delivery to the macula densa, thereby restoring tubuloglomerular feedback and inducing afferent arteriolar vasoconstriction (Pac et al., 2023; Kitada et al., 2020). This reduces intraglomerular pressure and hyperfiltration, a mechanism central to chronic renoprotection and potentially relevant to acute renal vulnerability as well (Table 1, Figure 1).

In HF, SGLT2is produce a distinctive form of decongestion characterized by preferential reduction of interstitial fluid with relatively limited depletion of effective intravascular volume (Pac et al., 2023; Voors et al., 2022). This profile may explain why they improve congestion without provoking the same degree of neurohormonal activation often seen with aggressive loop diuresis. Reduced preload, improved tissue edema, lower venous pressures, and more favorable renal perfusion may collectively interrupt the cycle of acute cardiorenal dysfunction.

These properties are particularly attractive in acute decompensated HF, where even modest relief of congestion can influence symptoms, renal course, and early rehospitalization. At the same time, hemodynamic benefit is highly phenotype-dependent. In patients with severe hypovolemia, unstable shock, or profound septic vasodilation, natriuretic effects may become undesirable or require careful timing and monitoring.

Thus, hemodynamic unloading should not be viewed merely as a diuretic effect, but rather as a coordinated modulation of cardiorenal coupling, intrarenal hemodynamics, tissue congestion, and neurohormonal stress.

### 3.5 Immunomodulation, inflammasome suppression, and endothelial barrier protection

Acute organ injury is strongly shaped by inflammatory amplification and microvascular dysfunction. In this context, SGLT2is have increasingly been recognized as modulators of innate immunity and endothelial stability.

One of the most frequently discussed pathways is suppression of the NLRP3 inflammasome, a central driver of sterile inflammation in ischemic and metabolic tissue injury (Lee and Riella, 2024; Kounatidis et al., 2023; Scisciola et al., 2022). Through reduced ROS generation, improved mitochondrial function, and increased β-hydroxybutyrate signaling, SGLT2is may decrease inflammasome activation and the downstream release of interleukin-1β and interleukin-18 (Lee and Riella, 2024; Scisciola et al., 2022). This mechanism is potentially relevant across the heart, kidney, brain, and lung (Table 1, Figure 1).

SGLT2is may also influence immune-cell phenotype. Several experimental studies suggest a shift of macrophages from a pro-inflammatory M1-like state toward a more reparative M2-like phenotype, which may favor inflammation resolution and tissue repair (Lee and Riella, 2024; Medeiros et al., 2025). Although this literature is still developing, it aligns well with the broader observation that SGLT2is reduce circulating inflammatory mediators such as C-reactive protein and interleukin-6 (Scisciola et al., 2022) (Table 1, Figure 1).

The vascular endothelium is another likely target. Endothelial dysfunction and glycocalyx degradation contribute to capillary leak, edema formation, coagulation abnormalities, and organ hypoperfusion in acute illness. Preclinical studies suggest that SGLT2is improve endothelial function and may help preserve glycocalyx integrity (Durante et al., 2021; Wang et al., 2025; Alshnbari et al., 2020). In principle, this could reduce microvascular permeability, stabilize the alveolar-capillary and glomerular barriers, and improve tissue oxygen delivery (Table 1, Figure 1).

Collectively, these immuno-endothelial actions provide an important bridge between cellular protection and organ-level outcomes. They suggest that SGLT2is may not simply alter one metabolic variable, but instead dampen the inflammatory and vascular propagation of acute injury.

## 4 Organ-specific evidence in acute injury

### 4.1 Acute heart failure

Among all acute clinical settings, the evidence for SGLT2is is strongest in AHF. These drugs have already become foundational therapies in chronic heart failure, and more recent trials indicate that they can also be initiated safely during or shortly after hospitalization for acute decompensation (Vaduganathan et al., 2022; Voors et al., 2022).

In the EMPULSE trial, empagliflozin initiated in stabilized patients hospitalized for AHF improved a hierarchical composite outcome including death, HF events, and quality of life (Voors et al., 2022). Similarly, the SOLOIST-WHF trial showed that sotagliflozin initiated before or shortly after discharge in patients with worsening HF reduced cardiovascular death and urgent heart failure-related events (Vaduganathan et al., 2022). These results are highly relevant because they demonstrate that SGLT2is can favorably alter early post-hospital trajectories rather than acting only as long-term disease modifiers (Table 2).

**TABLE 2:** Organ-specific evidence for acute protection by SGLT2 inhibitors.

Clinical setting	Representative studies	Main message	Dominant time window	Strength of evidence	Key limitation
Acute heart failure	EMPULSE, SOLOIST-WHF/meta-analyses	Early initiation after stabilization improves outcomes	Days	Strong	Not tested in severe shock phenotypes
Post-MI remodeling	EMMY, experimental MI models	May attenuate remodeling and improve biomarkers	Days to weeks	Moderate	Hard-event evidence limited
Acute kidney injury prevention	Perioperative observational data, mechanistic reviews	Hypothesis-generating	Days	Emerging	No dedicated AKI randomized controlled trials
Arrhythmias	DAPA-HF *post hoc*, EMPA-ICD	Reduced ventricular arrhythmic burden possible	Hours	Moderate	Outcome heterogeneity
Stroke/Brain injury	Meta-analyses, observational studies, preclinical review	Preclinical neuroprotection plausible, but randomized trials and meta-analyses show neutral stroke outcomes	Hours	Weak	No convincing reduction in stroke incidence in randomized evidence
ARDS/Acute lung injury	Mechanistic studies, indirect evidence	Purely speculative	Hours to days	Weak	No dedicated clinical trials
Acute liver injury	Post-authorization safety studies	Hepatic safety favorable; efficacy unproven	Days to weeks	Weak	Mostly safety rather than efficacy data

Strength of evidence integrates study design, reproducibility across independent studies, availability of randomized clinical data, consistency of clinically relevant outcomes, and degree of mechanistic validation.

The biological explanation is likely multifactorial. Preferential interstitial decongestion reduces pulmonary and systemic edema; restoration of tubuloglomerular feedback improves renal handling of sodium and water; metabolic effects may support myocardial efficiency; and anti-inflammatory as well as endothelial benefits may improve recovery after decompensation (Pac et al., 2023; Voors et al., 2022; Lee and Riella, 2024). Notably, benefit appears to extend across a wide spectrum of ejection fraction and is not limited to patients with diabetes (Vaduganathan et al., 2022; Wang and Isom, 2020; Anker et al., 2021; Zannad et al., 2020).

That said, current evidence supports initiation in hemodynamically stabilized patients rather than in those with overt cardiogenic shock or severe hypoperfusion. Thus, AHF represents the most mature and clinically actionable acute-use scenario for SGLT2is, but not necessarily a universal indication across all instability phenotypes.

### 4.2 Acute myocardial infarction and post-infarction remodeling

SGLT2is have not consistently been shown to reduce the incidence of MI itself in major outcome trials (Nall et al., 2024; Mukhopadhya et al., 2023). Therefore, their role in MI should not be framed primarily as anti-atherothrombotic prevention. Rather, the more compelling question is whether they reduce post-infarction injury progression, adverse ventricular remodeling, and transition to HF.

Experimental studies support this possibility. SGLT2is have been shown to attenuate oxidative stress, improve myocardial energetics, enhance autophagy, and limit forms of regulated cell death such as autosis, thereby reducing structural deterioration after infarction (Jiang et al., 2022; Zhong et al., 2023; Lahnwong et al., 2020; Buonpane et al., 2026). These findings align well with the broader mitochondrial and ionic stabilization pathways discussed above.

Clinical evidence has become more encouraging with the EMMY trial, in which early initiation of empagliflozin after acute MI led to greater reductions in NT-proBNP and favorable changes in cardiac structure and function (von et al., 2022). Although the trial was not powered for hard cardiovascular outcomes, it provides proof-of-concept that SGLT2is may beneficially influence early remodeling biology after infarction (Table 2).

This distinction is crucial: SGLT2is may not primarily prevent plaque rupture or coronary thrombosis, but they may mitigate the downstream cascade from acute ischemic injury to chronic heart failure. In translational terms, this makes them promising post-MI remodeling modifiers rather than anti-MI agents *per se*.

### 4.3 Acute kidney injury

The kidney is central to both the mechanism and clinical identity of SGLT2is. While their benefits in chronic kidney disease are well established, their role in acute kidney injury is still developing (Giorgino et al., 2020; Bailey et al., 2022).

Several mechanistic factors support acute renoprotection. By reducing proximal tubular transport work, SGLT2is decrease oxygen consumption and may alleviate cortical and medullary hypoxic stress (Giorgino et al., 2020; Bailey et al., 2022). Restoration of tubuloglomerular feedback lowers intraglomerular pressure, while favorable effects on venous congestion may improve renal hemodynamics in HF(4). In experimental ischemia-reperfusion models, SGLT2is have also been associated with reduced inflammation, fibrosis, microvascular congestion, and tubular injury (Song et al., 2025; Bailey et al., 2022).

Observational clinical data are encouraging. A recent study suggested that preoperative SGLT2i use in patients with type 2 diabetes undergoing major surgery was associated with a lower risk of postoperative AKI, particularly in high-risk subgroups (Song et al., 2025). Such findings raise the possibility of a preconditioning effect, whereby chronic SGLT2i therapy improves renal resilience before an anticipated insult (Table 2).

However, the evidence remains preliminary. Most clinical studies in AKI are observational and vulnerable to confounding. Moreover, the initial decline in estimated glomerular filtration rate after SGLT2i initiation can create uncertainty in acute settings. Accordingly, while the biological rationale for AKI protection is strong, dedicated randomized trials are still needed before firm recommendations can be made.

### 4.4 Arrhythmias as an expression of acute cardiac protection

Arrhythmias are common in HF, ischemic injury, and structural heart disease, and may represent both a consequence and a marker of acute myocardial stress. SGLT2is have attracted increasing interest because reduced arrhythmic burden may reflect a broader protective effect on myocardial physiology (Kolesnik et al., 2022).

In a *post hoc* analysis of DAPA-HF, dapagliflozin reduced the composite outcome of ventricular arrhythmias, resuscitated cardiac arrest, or sudden death (Curtain et al., 2021). Further support came from the prospective EMPA-ICD trial, which showed that empagliflozin reduced device-detected ventricular arrhythmia events in patients with type 2 diabetes carrying an ICD/CRT-D (Fujiki et al., 2024). Experimental studies also suggest attenuation of QT prolongation and reduced vulnerability to ischemia/reperfusion-induced ventricular arrhythmias (Özgür et al., 2021) (Table 2).

Mechanistically, these effects likely involve multiple pathways: improved intracellular sodium and calcium handling through NHE1-related mechanisms, suppression of late sodium current and CaMKII activity, improved mitochondrial function, reduction of oxidative stress, and lower fibrosis burden (Kolesnik et al., 2022; Mustroph et al., 2022; von Lewinski et al., 2022; Sato et al., 2023). Thus, the antiarrhythmic signal should not be viewed as a single ion-channel effect but as part of a broader network of myocardial stabilization.

Although more targeted arrhythmia trials are needed, current evidence suggests that SGLT2is may reduce electrical instability in selected high-risk populations and may therefore represent an important component of acute and subacute cardiac protection.

### 4.5 Brain injury and stroke

The relationship between SGLT2is and stroke remains complex. Mechanistically, these agents show several potentially beneficial actions in preclinical models of cerebral ischemia, including reduction in oxidative stress, attenuation of neuroinflammation, stabilization of the blood-brain barrier, and suppression of neuronal apoptosis (Lee and Riella, 2024; Al Hamed et al., 2020; Tsai et al., 2021). Such observations support a plausible neuroprotective role.

However, large randomized outcome trials have not consistently shown a reduction in stroke incidence (Mukhopadhya et al., 2023; Tsai et al., 2021; Butler et al., 2024). Meta-analyses of major cardiovascular outcome trials have generally yielded neutral results for stroke, even when overall cardiovascular outcomes improved (Mukhopadhya et al., 2023; Tsai et al., 2021). This implies that the major clinical benefits of SGLT2is are more closely tied to HF and cardiorenal pathways than to direct prevention of cerebrovascular events (Table 2).

By contrast, some real-world cohort studies have reported lower rates of new-onset stroke among SGLT2i users (Lin et al., 2022). However, these observational findings should be interpreted cautiously because they are susceptible to residual confounding and have not been confirmed in randomized trials or meta-analyses. At present, the overall clinical evidence does not support a meaningful reduction in stroke incidence with SGLT2is. Although preclinical neuroprotective mechanisms remain biologically plausible, large randomized trials and meta-analyses have consistently shown neutral stroke outcomes (Mukhopadhya et al., 2023; Tsai et al., 2021). Future studies should therefore focus less on stroke prevention *per se* and more on whether SGLT2is influence post-stroke injury severity, recovery, or neurovascular remodeling (Table 2).

### 4.6 Acute lung injury and acute respiratory distress syndrome

The rationale for evaluating SGLT2is in acute lung injury and ARDS arises from their anti-inflammatory, endothelial-protective, and decongestive properties (Alshnbari et al., 2020; Fernandez-Fernandez et al., 2020; Patoulias et al., 2021; Zimmermann et al., 2023). ARDS is characterized by alveolar-capillary barrier disruption, endothelial dysfunction, inflammatory amplification, and often concurrent injury to the heart and kidney (Bos and Ware, 2022). Because SGLT2is influence several of these pathophysiological axes, they have attracted translational interest.

Potential benefits may include suppression of cytokine-driven inflammation, preservation of endothelial function, reduction in microvascular permeability, and relief of pulmonary interstitial edema in patients with overlapping cardiorenal congestion (Lee and Riella, 2024; Durante et al., 2021; Alshnbari et al., 2020; Patoulias et al., 2021). These pathways were discussed extensively during the COVID-19 era, when endothelial injury and multi-organ dysfunction became central features of severe disease (Fernandez-Fernandez et al., 2020; Zimmermann et al., 2023; Ceriello et al., 2020; Varga et al., 2020; Madjid et al., 2020) (Table 2).

Nevertheless, no direct randomized or dedicated clinical trial evidence currently supports the use of SGLT2is in ARDS or acute lung injury. Most supporting data are indirect, derived from cardiovascular and renal studies, mechanistic reviews, or inflammatory biomarker analyses rather than dedicated ARDS trials (Fernandez-Fernandez et al., 2020; Patoulias et al., 2021; Zimmermann et al., 2023). As such, the current status of SGLT2is in acute lung injury should be regarded aspurely hypothesis-generating and speculative rather than clinically established (Table 2).

### 4.7 Acute liver injury and hepatic protection

Compared with the cardiorenal field, evidence for acute hepatic protection is substantially less developed. However, real-world safety analyses provide useful reassurance. Post-authorization studies have not shown increased risk of ALI with dapagliflozin or empagliflozin, and some analyses suggest numerically lower hepatic event rates compared with certain comparator therapies (Danysh et al., 2023; Rebordosa et al., 2024) (Table 2).

Mechanistically, SGLT2is may reduce hepatic stress indirectly by improving insulin resistance, decreasing body weight, lowering hepatic fat accumulation, and attenuating systemic inflammation and oxidative stress (Matuszewski et al., 2025; Rebordosa et al., 2024; Cho et al., 2024). These effects are best documented in chronic metabolic liver disease rather than in true acute liver injury syndromes. Experimental links to ferroptosis modulation are biologically interesting but still preliminary (Zhang et al., 2025; Wu et al., 2021).

Therefore, it is currently more accurate to conclude that SGLT2is exhibit a favorable hepatic safety profile with possible hepatoprotective signals, rather than established efficacy in ALI.

## 5 Translational perspective: from chronic disease drug to acute care strategy

The evidence reviewed above supports a conceptual transition: SGLT2is may be viewed not only as glucose-lowering agents or chronic cardiorenal therapies, but also as integrated modulators of acute organ stress responses. However, successful translation into acute care depends on careful attention to timing, patient selection, and safety.

The term “time-sensitive” acute protection refers broadly to interventions initiated within evolving phases of acute injury in which key pathophysiological processes remain modifiable. Importantly, these therapeutic windows likely differ substantially across organ systems and disease states. For example, Brain injury, stroke, and acute arrhythmias evolve most rapidly, with critical therapeutic windows occurring within hours of injury onset. In contrast, acute heart failure and acute kidney injury generally progress over days, representing an early phase of organ dysfunction during which intervention may be most effective. Acute respiratory distress syndrome (ARDS) occupies an intermediate position, typically evolving over hours to days as inflammatory and endothelial injury propagate. Post–myocardial infarction remodeling and acute liver injury generally extend over days to weeks and are characterized by ongoing repair, remodeling, and recovery processes (Vaduganathan et al., 2022; Voors et al., 2022; Song et al., 2025; von et al., 2022; Kolesnik et al., 2022; Al Hamed et al., 2020; Tsai et al., 2021; Butler et al., 2024; Bos and Ware, 2022; Rebordosa et al., 2024). Accordingly, SGLT2i-associated acute protection should not be interpreted as a single uniform therapeutic paradigm, but rather as a spectrum of temporally distinct intervention opportunities.

One useful framework is metabolic preconditioning. In high-risk patients already receiving SGLT2is before predictable stressors, such as major surgery or recurrent decompensated HF, chronic therapy may improve organ resilience by reducing tubular oxygen demand, enhancing mitochondrial quality control, and lowering inflammatory priming (Song et al., 2025; Bailey et al., 2022). This concept is appealing for AKI prevention and post-MI recovery, although it remains incompletely tested.

A second translational window is early post-stabilization initiation. The strongest example comes from AHF, where in-hospital introduction after hemodynamic stabilization improves outcomes (Vaduganathan et al., 2022; Voors et al., 2022). Post-MI remodeling may represent another such window, where injury propagation continues after the acute event but before chronic structural deterioration is fixed (Von et al., 2022; Butler et al., 2024). An important practical limitation, however, is that all currently approved SGLT2 is are administered orally, and virtually all available clinical evidence derives from chronic or subacute enteral dosing strategies. In critically ill patients who are nil per os, vomiting, mechanically ventilated, or receiving vasopressor support, gastrointestinal absorption may become unreliable (Tang et al., 2026). This raises an unresolved translational question as to whether current oral formulations are sufficient for true time-sensitive acute organ protection, particularly during the earliest phases of shock or severe systemic illness. At present, the most evidence-supported approach remains oral initiation after hemodynamic stabilization rather than during profound physiological instability. Future work may need to explore alternative delivery strategies, optimized timing windows, or potentially intravenous formulations if rapid-onset acute organ protection is ultimately pursued.

A third strategy is phenotype-guided deployment. Patients characterized by congestion, cardiorenal interaction, metabolic stress, endothelial dysfunction, or inflammatory activation may be more likely to benefit than those with profound shock, severe volume depletion, or rapidly evolving catabolism. Future studies may benefit from biomarker-guided enrichment strategies incorporating NT-proBNP, renal tubular injury markers, inflammatory mediators, oxidative stress indices, or endothelial injury markers. For example, elevated NT-proBNP levels may help identify patients with persistent hemodynamic congestion who are more likely to benefit from early decongestive effects, whereas renal tubular injury markers may better identify subclinical renal vulnerability before overt AKI develops. Similarly, inflammatory or endothelial dysfunction markers may help enrich patient subsets with heightened immune-endothelial activation in whom SGLT2i-mediated anti-inflammatory or vascular protective effects are more biologically relevant (Vaduganathan et al., 2022; Song et al., 2025; Bos and Ware, 2022).

In short, the most promising acute-care role for SGLT2is may not be at the point of maximal hemodynamic collapse, but rather in selected subacute and early recovery phases where key injury pathways remain active and treatment is better tolerated.

## 6 Safety considerations and practical boundaries

A balanced interpretation of SGLT2is in acute organ protection requires explicit recognition of safety limits.

### 6.1 Euglycemic diabetic ketoacidosis

The most important acute risk is euglycemic diabetic ketoacidosis (euDKA), particularly during prolonged fasting, perioperative stress, infection, reduced insulin administration, or low-carbohydrate intake (Wang and Isom, 2020). Because glucose levels may not be markedly elevated, diagnosis can be delayed. This risk is a major barrier to indiscriminate use in unstable acute-care settings.

### 6.2 Volume status and hemodynamic stability

Although SGLT2is often provide favorable decongestion, they can aggravate hypotension or prerenal physiology in patients who are already volume depleted or poorly perfused (Yang et al., 2025). Such concerns are especially relevant in severe sepsis, vasoplegic states, advanced cardiogenic shock, or aggressive combined diuretic strategies, where even modest natriuretic effects may worsen hemodynamic instability.

### 6.3 Renal function interpretation

The modest early decline in estimated glomerular filtration rate after SGLT2i initiation is usually considered a hemodynamic effect rather than nephrotoxicity in chronic care (Pac et al., 2023; Giorgino et al., 2020). In acute settings, however, this phenomenon may complicate interpretation, particularly when renal function is already unstable.

### 6.4 Perioperative management

Current perioperative practice often recommends temporary discontinuation to reduce ketoacidosis risk (Alguwaihes, 2021). This creates a practical tension: the same drugs that may theoretically precondition the kidney could also increase metabolic risk around surgery. Prospective studies are needed to refine interruption and reintroduction strategies.

### 6.5 Critical illness

Critically ill patients may also face increased susceptibility to genitourinary complications. Because SGLT2is increase glycosuria, concerns remain regarding genital infections and potentially complicated urinary tract infections, particularly in patients with prolonged urinary catheterization, severe illness, or impaired immune defenses (Danysh et al., 2023; Rebordosa et al., 2024). Although large meta-analyses suggest that the excess risk is driven mainly by genital mycotic infections rather than severe urinary tract infection overall, the safety profile in ICU populations remains insufficiently characterized.

Severe infection and multi-organ failure remain areas of uncertainty. Existing evidence does not support routine use of SGLT2is in broad intensive care populations, and careful patient selection will be essential if future acute-care applications are pursued.

### 6.6 Renal replacement therapy and advanced kidney failure

Evidence for SGLT2is in patients receiving renal replacement therapy remains extremely limited. Most major cardiovascular and renal outcome trials excluded patients undergoing dialysis or continuous renal replacement therapy, and therefore the efficacy and safety of SGLT2is in these settings are uncertain (Minutolo et al., 2026). In addition, reduced glycosuric effect in advanced kidney failure raises questions regarding the relevance of several proposed mechanisms in dialysis-dependent patients. Accordingly, extrapolation of acute organ-protective benefits to renal replacement therapy populations should be approached cautiously until dedicated studies become available.

## 7 Knowledge gaps and research priorities

Despite substantial progress, several major gaps remain. I have summarized the following limitations and prospects.

### 7.1 Lack of dedicated acute-injury trials

Most current evidence derives from chronic disease trials, *post hoc* analyses, or observational studies rather than studies specifically designed for acute injury endpoints.

### 7.2 Uncertain timing

It remains unclear whether SGLT2is are most effective before injury, during early stabilization, or in the remodeling phase.

### 7.3 Class effect versus agent-specific effect

Different SGLT2is vary in selectivity, half-life, and off-target pharmacology, and the clinical significance of these differences in acute injury is unknown.

### 7.4 Patient selection

Better definition of responsive phenotypes is needed, including distinctions by congestion status, ischemic burden, inflammatory profile, kidney reserve, and diabetes status.

### 7.5 Biomarker-guided strategies

Integration of cardiac, renal tubular, inflammatory, metabolic, and endothelial biomarkers may improve both mechanistic precision and trial design.

### 7.6 Evidence outside the cardiorenal axis

Stroke, ARDS, and ALI remain largely speculative indications and require more robust translational work.

### 7.7 Safety implementation science

Protocols for ketone monitoring, perioperative drug interruption, infection surveillance in critically ill patients, hemodynamic risk stratification, and safe reinitiation need prospective validation, particularly in ICU and renal replacement therapy populations.

## 8 Conclusion

SGLT2is should no longer be considered solely as glucose-lowering agents. A growing body of mechanistic and translational evidence indicates that they exert coordinated effects on mitochondrial quality control, cellular stress resistance, substrate utilization, hemodynamic unloading, inflammation, and endothelial integrity. These actions provide a biologically coherent basis for acute organ protection that is at least partly independent of glycemic control.

At present, the strongest evidence supports their use in AHF and early post-MI remodeling, with emerging promise in AKI prevention and arrhythmic risk reduction. By contrast, their roles in stroke, acute lung injury, and acute liver injury remain preliminary and should be interpreted cautiously.

Future work should move beyond general descriptions of pleiotropy and instead define time-sensitive therapeutic windows, responsive clinical phenotypes, and biomarker-informed safety frameworks. If these challenges can be addressed, SGLT2is may ultimately become a translational platform for organ protection that extends far beyond diabetes management.
